# Supplementary material for: Preparedness of Nursing Homes: A Typology and Analysis of Responses to the COVID-19 Crisis in a French Network
Source: Healthcare (Basel). 2024 Aug 30;12(17):1727. doi: 10.3390/healthcare12171727 (PMC11395430; doi:10.3390/healthcare12171727)
Supplement: Supplementary file 1 [file healthcare-12-01727-s001.zip › supplementary_01.pdf]

Table S1. The elderly care sector in France in 2019. Data from DREES (Ministry of Health), EHPA 2015 survey.

As of December 31, 2019, nearly 11,000 accommodation structures for the elderly in France provided around 770,000 places. This capacity has been increasing since 2007, with a notable 10% rise in autonomy residences between 2015 and 2019.

Types of Facilities:

- **EHPAD** (*Etablissements d'Hébergement pour Personnes Agées Dépendantes*): Represent 70% of these structures (n = 7,400), offering 79% of the available places. These facilities have the highest average capacity with 81 places per establishment.
    - **Public EHPADs**: 3,344 facilities (45.2%), typically larger with more extensive services.
    - **Private EHPADs**: 4,056 facilities (54.8%), providing a significant portion of the total accommodation.
- ***Résidences Autonomie***: Intermediate housing solutions with around 52 places on average.
- **EHPA** (*Etablissements d'Hébergement pour Personnes Agées*): Non-medicalized collective housing for relatively autonomous elderly, with about 21 places on average.
- **USLD** (*Unités de Soins de Longue Durée*): Attached to hospitals, providing long-term care for highly dependent elderly individuals.

In our study, the scope covers EHPADs, translated by nursing homes (NHs). The 290 private EHPADs (NHs) in the group therefore represent 3.9% of all EHPADs (NHs) in France and 7% of all private EHPADs (NHs).

Geographical Distribution:

- **Urban vs. Rural**: The availability of places varies significantly between urban and rural areas. Urban areas generally have higher availability and more extensive services. In contrast, rural areas, particularly in the northern and western parts of France, have fewer facilities.
- **Regional Disparities**: The northern and western regions generally have higher rates of accommodation, while the DROM (Overseas Departments and Regions), Corsica, and the southeastern quarter have fewer places. For instance, some departments have less than 80 places per 1,000 elderly residents, while others have over 170 places per 1,000.

Development Trends:

Significant growth in temporary accommodation and specific living units for residents with behavioral issues, such as Alzheimer's. The number of EHPADs with specialized care units (PASA and UHR) has also increased.

Financial Aspects:

Daily accommodation rates have slightly increased, reaching an average of €55.90 per day by the end of 2015. Rates are higher in private for-profit EHPADs compared to public ones.

Staffing:

Over 500,000 individuals were employed in these facilities by the end of 2015, with women representing 87% of the workforce. The staffing ratio varies, with higher ratios in more medicalized structures like USLDs.

Resident Demographics:

Residents are getting older, with an increase in the average age and dependency levels. As of 2015, half of the residents were over 87 years old, and a significant proportion were highly dependent.
