# Supplementary material for: Preparedness of Nursing Homes: A Typology and Analysis of Responses to the COVID-19 Crisis in a French Network
Source: Healthcare (Basel). 2024 Aug 30;12(17):1727. doi: 10.3390/healthcare12171727 (PMC11395430; doi:10.3390/healthcare12171727)
Supplement: Supplementary file 1 [file healthcare-12-01727-s001.zip › supplementary_03.pdf]

**Table S2. Correlation of the active variables with the first 4 components of the MCA**

| | First component (16.3%) | Second component (12.6%) | Third component (10.6%) | Fourth component (8.2%) |
|---|---|---|---|---|
| Number of accommodation beds | 0,107 | 0,162 | 0,259 | 0,057 |
| Presence of a protected living unit | 0,006 | 0,016 | 0,123 | 0,360 |
| Presence of a hospital emergency unit in the municipality | 0,217 | 0,367 | 0,077 | 0,036 |
| Level of primary care territorial structuring (municipality level) | 0,446 | 0,106 | 0,071 | 0,311 |
| Number of accommodation places per 1,000 people aged 75 and over in the county | 0,612 | 0,446 | 0,280 | 0,008 |
| Percentage of the people aged 75 and over in the county living in a nursing home | 0,636 | 0,334 | 0,421 | 0,002 |
| Percentage of people aged 75 and over in the total population of the county | 0,250 | 0,049 | 0,152 | 0,201 |
| Urban or rural character of the county | 0,001 | 0,279 | 0,105 | 0,167 |
