# Supplementary material for: Preparedness of Nursing Homes: A Typology and Analysis of Responses to the COVID-19 Crisis in a French Network
Source: Healthcare (Basel). 2024 Aug 30;12(17):1727. doi: 10.3390/healthcare12171727 (PMC11395430; doi:10.3390/healthcare12171727)
Supplement: Supplementary file 1 [file healthcare-12-01727-s001.zip › supplementary_04.pdf]

Table S3. Mortality and satisfying hospitalization requests among the respondents nursing homes from the French network, during the first wave of the COVID-19 outbreak, by clusters of nursing homes and the magnitude of the outbreak. French COVID-19 Nursing homes survey, 2020.

n (%)	**All**	**Cluster 1**	**Cluster 2**	**Cluster 3**	***p-value*** [*]
**Magnitude of the outbreak in the county: "High"**	**45**	**16**	**23**	**6**	-
COVID-19 mortality					*< 0.05*
At least 1 death	42 (93.3)	16 (100)	22 (95.7)	4 (66.7)	
No deaths	3 (6.7)	0 (0)	1 (4.3)	2 (33.3)	
Satisfying hospitalization requests for COVID-19[*]					*< 0.05*
No requests	2 (6.2)	0 (0)	1 (6.2)	1 (20.0)	
Requests generally satisfied	22 (68.8)	11 (100)	8 (50.0)	3 (60.0)	
Requests generally unsatisfied	8 (25.0)	0 (0)	7 (43.8)	1 (20.0)	
**Magnitude of the outbreak in the county: "High" & "Medium"**	**112**	**35**	**48**	**29**	-
COVID-19 mortality					*< 0.01*
At least 1 death	79 (70.5)	29 (82.9)	37 (77.1)	13 (44.8)	
No deaths	33 (29.5)	6 (17.1)	11 (22.9)	16 (55.2)	
Satisfying hospitalization requests for COVID-19*					*< 0.01*
No requests	20 (26.7)	4 (16.7)	7 (21.9)	9 (47.4)	
Requests generally satisfied	46 (61.3)	20 (83.3)	17 (53.1)	9 (47.4)	
Requests generally unsatisfied	9 (12.0)	0 (0)	8 (25.0)	1 (5.2)	

[*] Only data directly reported by the nursing homes responding to the survey were available.
