# Supplementary figures and images for: Preparedness of Nursing Homes: A Typology and Analysis of Responses to the COVID-19 Crisis in a French Network
Source: Healthcare (Basel). 2024 Aug 30;12(17):1727. doi: 10.3390/healthcare12171727 (PMC11395430; doi:10.3390/healthcare12171727)

**Figure S1. Scree plot**

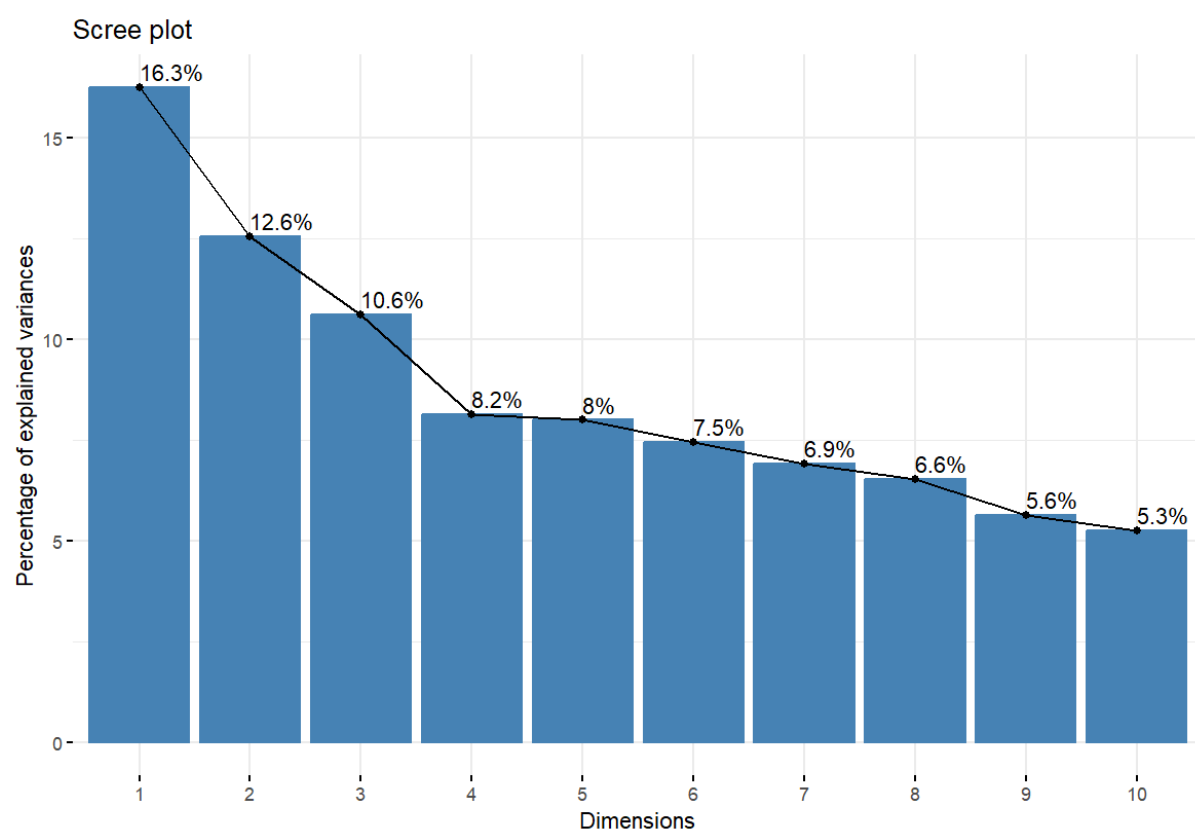

Supplement: Supplementary file 1 [file healthcare-12-01727-s001.zip › supplementary_02.pdf]
